# Classification of α-Helical Membrane Proteins Using Predicted Helix Architectures

**DOI:** 10.1371/journal.pone.0077491

**Published:** 2013-10-25

**Authors:** Sindy Neumann, Angelika Fuchs, Barbara Hummel, Dmitrij Frishman

**Affiliations:** 1 Department of Genome Oriented Bioinformatics, Technische Universität München, Wissenschaftszentrum Weihenstephan, Freising, Germany; 2 pRED, Pharma Research and Early Development, pRED Informatics, Roche Diagnostics GmbH, Penzberg, Germany; 3 Department of Urology/Women’s Hospital and Center for Clinical Research, University of Freiburg Medical Center, Freiburg, Germany; 4 Helmholtz Center Munich - German Research Center for Environmental Health (GmbH), Institute of Bioinformatics and Systems Biology, Neuherberg, Germany; University of South Florida College of Medicine, United States of America

## Abstract

Despite significant methodological advances in protein structure determination high-resolution structures of membrane proteins are still rare, leaving sequence-based predictions as the only option for exploring the structural variability of membrane proteins at large scale. Here, a new structural classification approach for α-helical membrane proteins is introduced based on the similarity of predicted helix interaction patterns. Its application to proteins with known 3D structure showed that it is able to reliably detect structurally similar proteins even in the absence of any sequence similarity, reproducing the SCOP and CATH classifications with a sensitivity of 65% at a specificity of 90%. We applied the new approach to enhance our comprehensive structural classification of α-helical membrane proteins (CAMPS), which is primarily based on sequence and topology similarity, in order to find protein clusters that describe the same fold in the absence of sequence similarity. The total of 151 helix architectures were delineated for proteins with more than four transmembrane segments. Interestingly, we observed that proteins with 8 and more transmembrane helices correspond to fewer different architectures than proteins with up to 7 helices, suggesting that in large membrane proteins the evolutionary tendency to re-use already available folds is more pronounced.

## Introduction

Since the determination of the first membrane protein structure in 1985 [1] a lot has changed in our knowledge about the membrane protein structure space. Initially, α–helical membrane proteins appeared to adopt a very simple architecture with transmembrane helices oriented more or less perpendicular to the membrane plane forming an α-helix bundle [2], [3]. However, recent structures have shown that they can be much more complex [4]–[10], warranting research on the structural variability of membrane proteins by means of structure classification methods.

Classification approaches, such as SCOP [11] (Structural Classification Of Proteins) and CATH [12] (Class, Architecture, Topology, Homologous superfamily), aim at exploring the full diversity of protein structures by enumerating and organizing all existing protein architectures. However, our comparative analysis of the structural classification of α-helical membrane proteins in these databases has shown that the fold definition initially developed for globular proteins is applicable to membrane proteins only to a limited degree [13]. We therefore suggested revising the fold definition for membrane proteins by incorporating more fine-grained structural features such as helix-helix interactions.

Current structural classification approaches specifically tailored to membrane proteins include methods using helix interaction patterns [14] and sequence information combined with topology conservation [15]. The former method classifies known 3D structures of membrane proteins according to the similarities of their helix interaction graphs and, in spite of its simplicity, is able to reconstruct structural classification of SCOP and CATH nearly perfectly [14]. In contrast, the latter approach is solely based on sequence information and predicted structural features. Proteins are classified according to similarities in their amino acid sequence and their topology (i.e. the number of transmembrane helices and loop lengths). Both approaches have their advantages and disadvantages. The strength of the first method is that structural similarities between proteins sharing no sequence similarity (often resulting from convergent evolution) can be revealed while the second method allows for a more comprehensive classification of membrane proteins. Therefore, it seems desirable to combine both approaches in order to exploit their full potential.

Here, we present an alternative version of our structural classification approach initially developed in 2010 [14]. While in the previous version helix interaction graphs were directly obtained from three-dimensional membrane protein structures we now predict these graphs from sequence. This methodology is applied to provide an exhaustive structural classification of membrane protein families available in the CAMPS database [15] and to identify protein clusters with similar helix interaction patterns that are not related at the sequence level. While certainly not meant to replace elaborate classification systems based on known atomic coordinates our approach represents a useful tool for exploring those parts of the membrane protein universe that are not yet covered by 3D structural information and for rational selection of targets for structural genomics projects.

## Materials and Methods

### Evaluation Dataset

A dataset of α-helical membrane proteins with available 3D structures was used to test how well structural similarities between membrane proteins can be reproduced from predicted helix architectures. All protein chains with more than four annotated transmembrane helices were obtained from PDBTM [16] together with their topology information as derived with the TMDET algorithm [17]. Proteins with fewer transmembrane helices were omitted as they lack the required diversity of possible helix interaction arrangements [14]. Sequence redundancy among all obtained protein chains was removed at the 95% identity level, yielding a dataset of 152 proteins which will be further referred to as PDB_TEST.

Within this dataset the number of observed transmembrane helices varied between 5 and 13, with seven transmembrane helices being the most prevalent number found in 59 protein chains. SCOP [11] and/or CATH [12] annotations could be obtained for 54 chains although only 24 chains had a classification in both databases. In accordance with the current approach adopted both by SCOP and CATH for transmembrane proteins [13], all proteins were treated as single domain proteins.

### Classification Dataset

The analysis of the predicted membrane protein fold space was conducted for a comprehensive set of clusters of structurally related α-helical membrane proteins (so called structurally correlated (SC) clusters) [15] as available from the CAMPS 2.0 database. For each of the 1353 SC-clusters we defined the most common number of transmembrane helices (TMHs) among the members (further referred to as the representative TMH number) and selected those SC-clusters with a representative TMH number of at least five and a structural homogeneity (reflecting the variation of the TMH number within the cluster, see [18]) of at least 0.80. The latter parameter was used to ensure that those proteins having the representative TMH number indeed represent the large majority of the corresponding cluster. From each of the 431 SC-clusters satisfying the above conditions the 50 most divergent protein members (according to sequence identity) with a TMH number equaling the representative TMH number of the corresponding SC-cluster were retained for further consideration. In case fewer than 50 members were available, all of them were selected. This procedure resulted in a final dataset containing 14,917 membrane protein sequences further referred to as CAMPS_SC.

### Prediction of Individual Helix Architectures for the Evaluation Dataset

Helix architectures were derived for all proteins in the evaluation dataset using helix-helix contacts predicted by TMHcon [19]. Briefly, TMHcon is a neural network-based residue contact predictor incorporating general and membrane protein-specific input features. Two versions of the TMHcon predictor were employed (see reference [19] for more details): i) NN4, considering all residue contacts between any pair of transmembrane helices, and ii) NN4-D, considering only long-range residue contacts between sequentially non-adjacent helix pairs.

After predicting residue contacts with TMHcon, helix interactions were derived from these contacts by first selecting a subset of residue contacts according to a fitted contact formula describing the expected number of contacts for a certain number of transmembrane residues [19]. For both networks separately (NN4 and NN4-D), a minimum number of contacts *C* for a given helix pair was required to identify interacting helices. In agreement with earlier results [19], thresholds of *C* = 9 (NN4) and *C* = 15 (NN4-D) were selected for predicting helix architectures as these thresholds were shown to optimize sensitivity and specificity of the resulting prediction. The final set of interacting helices was obtained by combining the predicted helix interactions of both neural networks.

### Prediction of Consensus Helix Architectures for the Classification Dataset

For the classification dataset a representative helix architecture was obtained for each SC-cluster by first predicting individual helix architectures for each protein in the cluster and then combining those individual architectures into a consensus architecture. During the consensus prediction step all helix interactions occurring in more individual architectures than a pre-set consensus threshold (*con*) were transferred to the consensus architecture.

### Benchmark Consensus Dataset

As the required stringency of the consensus threshold depends on the sensitivity and selectivity of the preceding helix interaction prediction, optimal values for the minimum number of required contacts *C* for NN4 and NN4-D of TMHcon and the consensus threshold *con* had to be newly re-determined using a subset of all clusters in the classification dataset with a known PDB [20] structure. This benchmark dataset was derived by searching all protein sequences contained in these SC-clusters against PDBTM [16] (version 2.3) using BLAST [21] for matches with at least 95% sequence identity and at least 95% sequence coverage. Theoretical models and structures with a resolution worse than 4 Å were ignored. Furthermore, only structures were considered whose TMH number (according to TOPDB [22] or PDBTM, if the protein was not available in TOPDB) corresponds to the representative TMH number of the respective SC-cluster. If several structures were available for one SC-cluster the structure with the best resolution was chosen. Twenty-eight SC-clusters were found to be associated with a known structure and those clusters together with the 28 PDB proteins representing these SC-clusters will be further referred to as CAMPS_TEST.

### Benchmarking Consensus Helix Architecture Quality

To evaluate the quality of the predicted consensus helix architectures true helix interaction graphs were obtained for the structures in CAMPS_TEST by considering all TMH pairs (TMH annotations were taken from TOPDB/PDBTM) with at least one helix-helix contact as interacting. A helix-helix contact was defined as a residue pair (located on different TMHs) having a spatial distance of less than 5.5 Å between any non-hydrogen atoms.

Consensus helix interaction graphs were predicted for each SC-cluster in CAMPS_TEST using varying values for the contact thresholds for NN4 and NN4-D and the consensus threshold *con*. For each set of thresholds sensitivity and specificity of the final consensus helix architectures were calculated in comparison to the true helix interaction graphs. Sensitivity was defined as the proportion of all interacting helices in the true helix architectures that were also present in the predicted consensus architectures while specificity described the proportion of all true non-interacting helices that were also absent in the consensus architectures. Furthermore, consensus architectures were also compared to helix architectures predicted with TMHcon for individual cluster proteins using default thresholds (*C* = 9 and *C* = 15 for NN4 and NN4-D, respectively) and both sensitivity and specificity were determined analogously.

### Predicting Final Consensus Helix Architectures

Using the combination of thresholds for NN4, NN4-D and *con* with the best sensitivity at a given specificity (see Results and Discussion), consensus architectures were generated for all 431 SC-clusters in CAMPS_SC.

### Evaluation of HISS-based Classifications Using Predicted Helix Architectures

Similarities between predicted helix architectures were quantified using the HISS (Helix Interaction Similarity Score) scoring system [14] which tests for common helix interactions between two membrane proteins by quantifying the fraction of helix interactions present in both structures relative to the total number of helix interactions observed in the individual proteins. Helix interactions can be weighted differently depending on the number of residue contacts and on the sequential distance between two helices.

To determine the best method for distinguishing proteins with similar helix architecture from those with different helix architecture even when only a fraction of all observed helix interactions are predicted while other interactions are wrongly predicted, HISS scores [14] were calculated for all protein pairs of the evaluation dataset having the same number of transmembrane helices and a consistent annotation (‘same fold’ *versus* ‘different fold’) in SCOP and CATH. Different variations of HISS scores were tested where all predicted helix interactions were either treated equally (‘unweighted strategy’) or helix interactions with more than 15 predicted helix contacts were upweighted with a factor 1.5 (‘weighted strategy’). Both approaches were compared to the SCOP/CATH classification using a receiver operator characteristic (ROC) curve which plots the achieved true positive rate against the false positive rate over the full range of possible HISS score thresholds, with any point above the diagonal corresponding to a better prediction than random. The overall classification quality was quantified using the Area Under the Curve (AUC) measure. ROC curve and AUC calculations were executed with R using the ROCR package [23]. Furthermore, for both weighted and unweighted HISS scores, individual classifications were derived where all proteins satisfying a specified HISS score threshold were classified to the same fold. Then sensitivity and specificity of this classification with respect to the SCOP/CATH reference classification were calculated. Sensitivity is defined as the fraction of all protein pairs with the same SCOP/CATH fold annotation that satisfied the specified HISS score threshold. Specificity is defined as the fraction of all protein pairs with different SCOP/CATH fold annotation and HISS score below the specified threshold.

Within a second evaluation step HISS scores (‘weighted’ and ‘unweighted’) were calculated for *all* proteins of the evaluation dataset having the same number of transmembrane helices irrespective of their presence in SCOP and/or CATH. Protein pairs satisfying pre-defined HISS score thresholds were clustered with the MCL algorithm [24] using default parameters. The resulting classifications were compared to the previously derived classification of helix architectures using known membrane protein structures as available from HISSdb [14]. Again, the AUC measure and sensitivity/specificity of individual classifications were calculated to quantify the classification success. This time, sensitivity describes the fraction of all protein pairs belonging to the same HISSdb architecture that were also found in the same MCL cluster based on predicted helix interaction while specificity quantifies the fraction of all protein pairs classified to different MCL clusters in the prediction-based classification out of all protein pairs classified to different architectures within HISSdb.

### Large-scale Classification of Consensus Helix Architectures

We conducted a comparison of all consensus architectures generated for the SC-clusters in CAMPS_SC using the HISS scoring system [14], considering only pairs of consensus architectures with the same representative TMH number. All HISS scores above different pre-defined thresholds were used to cluster the consensus architectures using the MCL algorithm [24] with varying inflation values. As the inflation value is an MCL parameter that controls the granularity of the clustering (the higher the value the more fine-grained the clustering), different combinations of the two parameters (HISS score threshold, inflation value) were tested and the final MCL clusters (termed HIS ( = helix interaction similarity) clusters) were validated using the Pfam-A [25] annotations of the corresponding proteins. If a protein was classified to a Pfam-A family having a clan assignment, then the clan annotation was considered, otherwise the family annotation was used. Sensitivity was defined as the fraction of all proteins covered by the HIS clusters with the same Pfam-A annotation that were also found in the same HIS cluster. Similarly, specificity was calculated as the fraction of all proteins with different Pfam-A annotations that were also found in different HIS clusters. The parameter combination with the best sensitivity at a given specificity was chosen for the final set of clustered consensus architectures.

### Gene Ontology Enrichment Analysis

To identify significantly enriched Gene Ontology [26] (GO) terms within the set of proteins covered by the HIS clusters, we used the Ontologizer software [27]. The software requires a GO ontology file, an annotation file (with GO terms mapped to genes), so-called study sets (genes/proteins of interest), and a population set (reference set) as input. We chose the GO slim [28] generic ontology (OBO v1.2; as of January 11, 2012) which is a subset of the whole GO containing high-level terms and the unfiltered UniProt annotation file (as of December 13, 2011; both files were downloaded from http://www.geneontology.org). Two separate enrichment analyses were conducted. In the first analysis (further referred to as protein class level enrichment analysis) all membrane proteins covered by the HIS clusters were grouped according to their TMH number and each group constituted a study set resulting in eleven study sets (5–15 TMHs). The union of all eleven study sets formed the population set. In the second analysis (further referred to as cluster level enrichment analysis) each HIS cluster itself described a study set using those members whose TMH number corresponded to the representative TMH number of the corresponding cluster, yielding 151 study sets. The population set was again the sum of all study sets. For further consideration, we selected all enriched GO terms returned by Ontologizer with an adjusted *P*-value better than or equal to 0.05. The adjusted *P*-values were calculated using the Bonferroni correction method (which is one of the optional parameters of the Ontologizer software).

### Availability

Consensus helix interaction graphs and the clustering of SC-clusters into HIS clusters can be downloaded at http://webclu.bio.wzw.tum.de/CAMPS2.0/download.jsp.

## Results and Discussion

### Overview of the Methodology

In this study we sought to further improve large-scale classification of predicted membrane protein architectures by introducing a new classification level in our CAMPS database [15] that goes beyond similarity of amino acid sequence and predicted transmembrane topology. To this end, predicted helix-helix interactions were used to identify, for each CAMPS cluster, a consensus helix interaction architecture representing common helix interactions among all cluster members and the similarity among these consensus helix interaction patterns was subsequently used to group initially separate CAMPS clusters.

**Figure 1:**
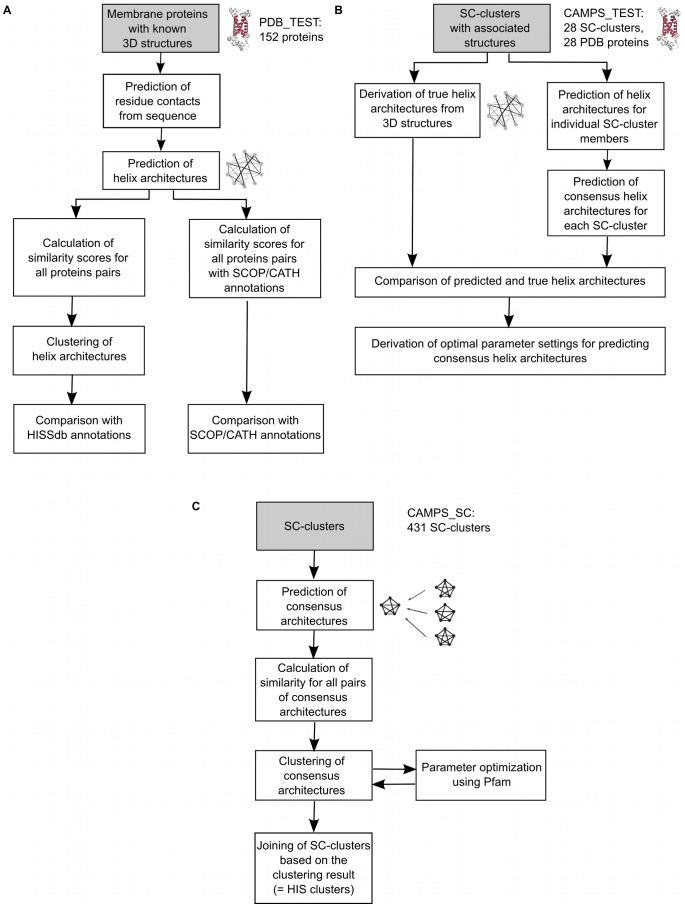
Overview of the methodology. A: Classification of α-helical membrane protein structures using predicted helix architectures. B: Parameter optimization for generating consensus helix architectures. C: Classification of α-helical membrane proteins using consensus helix architectures.

To optimize and analyze the quality of this new classification procedure the two major components of the classification process were separately evaluated. To test how well predicted helix interactions can be used to identify structural similarities among membrane proteins, helix interaction architectures were predicted for individual sequences with known three-dimensional structures. Then these architectures were classified based on their similarity. The obtained classification was compared to the corresponding SCOP/CATH classification of the same proteins as well as to our own HISSdb classification (dataset PDB_TEST, Figure 1A). At the cluster level consensus helix architectures were first derived only for those CAMPS clusters, where a representative PDB structure was available. Subsequently, the quality of those consensus helix architectures in comparison to the true helix interaction patterns, derived from the PDB structures, was analyzed (dataset CAMPS_TEST, Figure 1B).

Finally, the complete process of deriving consensus helix architectures and classifying them based on helix interaction similarity was applied to a comprehensive set of CAMPS clusters (dataset CAMPS_SC, Figure 1C) and the resulting set of predicted membrane protein architectures was analyzed.

### Classification of Predicted Helix Architectures in Comparison to SCOP and CATH

The similarity of predicted helix interaction graphs and the possibility of discriminating proteins with similar and different architectures based on these graphs was first evaluated using proteins with available 3D structure that are classified consistently in SCOP and CATH either to the same fold or to different folds. As four helix bundle proteins are known to pose a problem to structural classification in general [13], only proteins with at least five transmembrane helices were considered. The resulting test set contained 54 protein chains forming 211 protein pairs of which 95 had the same fold assignment in SCOP/CATH while the remaining protein pairs had the same number of transmembrane helices but different fold assignments. Helix interactions were predicted for all proteins based on helix-helix contacts obtained with TMHcon [19] using a two step filtering procedure where a large set of residue contacts is selected in the first step but only those helix pairs are predicted as interacting that make at least *C* residue contacts (see Materials and Methods). Similarities among these predicted helix interactions were quantified using HISS similarity scores [14] in two variations: i) treating all predicted helix interactions equally, and ii) upweighting interactions with many predicted contacts.

As seen in Table 1, proteins from the same fold in SCOP and CATH consistently have higher HISS scores than proteins from different folds independent of the HISS calculation method applied. Accordingly, the classification of proteins into “same” or “different” fold based on HISS scores results in a classification well above random as can be concluded from the reported AUC (‘area under the ROC curve’) values which were found to be as high as 0.848 (a random prediction would result in an AUC value of 0.5). The incorporation of weights for HISS score calculation had no major effect on the prediction (AUC of 0.846 without weights compared to 0.848 with weights).

**Table 1:** Classification of proteins in SCOP and CATH using predicted helix interactions.

HISS^a^	Avg(HISS)_same_ b	Avg(HISS)_diff_ c	AUC^d^	Score^e^	Sensitivity^f^ [%]	Specificity^g^ [%]
uw	0.890	0.749	0.846	0.88	72.6	78.4
				0.90	65.3	92.2
w	0.864	0.665	0.848	0.82	76.8	81.0
				0.88	53.7	91.4

Helix interactions were predicted using the threshold combination C = 9 (network NN4) and C = 15 (network NN4-D), see Materials and Methods. HISS scores were calculated with and without weighting edges.

a HISS scores were calculated both without weighting helix interactions (uw) and with up weighting interactions involving >15 residue contacts by a factor 1.5 (w).

b Avg(HISS)_same_: average HISS score for proteins classified to the same fold in SCOP and CATH.

c Avg(HISS)_diff_: average HISS score for proteins classified to different folds in SCOP and CATH.

d AUC: area under the curve describing how well proteins with the same fold can be differentiated from proteins with different folds (AUC = 0.5 would correspond to a random prediction).

e Score: HISS score threshold used to identify proteins with the same helix architecture. For both weighted and unweighted HISS scores, two thresholds were chosen such that the specificity of the obtained classifications most closely approached either 80% or 90%.

f Sensitivity: Fraction of all protein pairs with the same SCOP/CATH fold annotation having a HISS score above the specified threshold.

g Specificity: Fraction of all protein pairs with different SCOP/CATH fold annotation having a HISS score below the specified threshold.

Using specific HISS score thresholds to predict proteins as belonging to the same fold (Table 1), the sensitivity and specificity of such a prediction can be calculated by testing how many proteins that actually belong to the same fold satisfy this threshold and how many proteins that are classified to separate folds in SCOP and CATH have HISS scores below this threshold. The lower this score threshold is chosen, the more sensitive is such a prediction at the cost of reduced specificity. Aiming at a specificity of 80%, the best prediction was obtained with weighted HISS scores with a sensitivity of nearly 77%. Similarly, the best prediction with 90% specificity (unweighted HISS scores) resulted in a sensitivity of 65%.

These results are highly encouraging with respect to the structural classification of membrane proteins. Of course, similar structures can not be identified with equal quality as based on known structures, where a HISS based classification resembles SCOP and CATH nearly perfectly with an AUC value of 0.998 [14]. Still, a large fraction of all proteins having the same helix architecture can be recognized with high specificity. Importantly, this similarity can also be determined using predicted helix interactions when the sequence similarity between the analyzed proteins is too low to confidently assign a common fold. For example, bovine rhodopsin (PDB 1GZM, chain A) was found to have HISS scores ≥0.9 with several other rhodopsins such as halorhodopsin (PDB 1E12, chain A) or sensory rhodopsin (PDB 1XIO, chain A) although the sequence similarity among these proteins is too low to obtain a proper sequence alignment (for comparison: proteins with identical helix interactions result in HISS = 1.0).

### Classification of Predicted Helix Architectures in Comparison to HISSdb

As SCOP and CATH both cover only a fraction of all available membrane protein structures, we used our own previously introduced classification of known membrane protein structures called HISSdb [14] to test the classification of predicted helix architectures on the full set of PDB proteins. HISS scores (weighted and unweighted) were calculated for *all* protein pairs having the same number of transmembrane helices within our evaluation dataset (2520 protein pairs in total of which 1540 were classified within HISSdb to the same architecture). Following the standard HISSdb construction protocol the MCL clustering algorithm with default parameters was used to obtain clusters of proteins having highly similar helix interaction patterns and the obtained clusters were compared to the original HISSdb clusters (Table 2).

**Table 2:** Classification of all proteins with solved 3D structure using predicted helix architectures in comparison to the HISSdb database.

HISS^a^	Avg(HISS)_same_ b	Avg(HISS)_diff_ c	AUC^d^	Score^e^	Sensitivity^f^ [%]	Specificity^g^ [%]
Uw	0.812	0.717	0.704	0.89	77.6	74.5
				0.90	37.9	85.3
W	0.775	0.622	0.752	0.85	66.0	81.0
				0.88	48.7	90.5

Helix interactions were predicted using the threshold combination C = 9 (network NN4) and C = 15 (network NN4-D), see Materials and Methods. HISS scores were calculated with and without edge weighting. Final classifications were obtained by clustering all proteins satisfying the specified HISS score thresholds using the MCL algorithm.

a HISS scores were calculated both without weighting helix interactions (uw) and with up weighting interactions involving >15 residue contacts by a factor 1.5 (w).

b Avg(HISS)_same_: average HISS score for proteins classified to the same helix architecture type in HISSdb.

c Avg(HISS)_diff_: average HISS score for proteins classified to different helix architecture types in HISSdb.

d AUC: area under the curve describing how well proteins with the same fold can be differentiated from proteins with different folds (AUC = 0.5 would correspond to a random prediction).

e Score: HISS score threshold used for clustering proteins with the MCL algorithm. For both weighted and unweighted HISS scores, two thresholds were selected such that the specificity of the obtained classifications most closely approached either 80% or 90%.

f Sensitivity: Fraction of all protein pairs with the same HISSdb architecture annotation assigned to the same MCL cluster.

g Specificity: Fraction of all protein pairs with different HISSdb architecture annotation assigned to different MCL clusters.

Again, protein pairs belonging to the same HISSdb cluster were found to have significantly higher HISS scores than proteins from different HISSdb clusters based on both weighted and unweighted HISS scores (Table 2). In contrast to the SCOP/CATH analysis, however, this time weighted HISS scores were able to reproduce the original HISSdb classification more closely than unweighted HISS scores as indicated by the obtained AUC values (0.752 with weighted and 0.704 with unweighted scores). Importantly, both AUC values are again clearly higher than random, indicating that predicted helix interactions are sufficiently accurate to identify structural similarities among the full set of membrane proteins with solved 3D structure. However, it must be noted that classifications using the full evaluation dataset have lower AUC values than the subset of SCOP/CATH proteins discussed earlier. As the latter dataset contains roughly only 10% of all protein pairs from the full evaluation dataset, an AUC value of 0.75 (as obtained from a much larger set of HISSdb structures with weighted HISS scores) seems to be a more realistic estimate of what is optimally achievable with predicted helix interactions than the AUC value of 0.85 reported for SCOP/CATH proteins.

After clustering all protein pairs satisfying a given HISS score threshold with the MCL algorithm, we evaluated the sensitivity and specificity of the resulting classifications with respect to the original HISSdb classification (Table 2). In this case sensitivity describes the fraction of all protein pairs belonging to the same HISSdb cluster that were also clustered together with the MCL algorithm based on predicted helix interaction. Similarly, specificity describes the fraction of all protein pairs classified to different clusters in the prediction-based classification out of all protein pairs classified to different folds in the original structure-based classification. Using weighted HISS scores, the HISSdb classification of transmembrane proteins with known 3D structures could be reproduced with a specificity of 81% and a sensitivity of 66%. Even at the specificity level of 90% nearly 50% (48.7%) of all proteins with the same HISSdb clusters were also correctly identified to have similar folds based on predicted helix architectures. As already indicated by AUC values, unweighted HISS scores are not able to express similarities and differences of predicted helix architectures as well as weighted HISS scores. As can be seen from Table 2, a minor change in the clustering score threshold from 0.89 to 0.90 leads to significantly deviating classifications with increased specificity from 74.5% to 85.3% accompanied by an even stronger decrease in sensitivity from 77.6% to 37.9%. We are therefore not able to report sensitivity and specificity for classifications with close to 80% or 90% specificity as we did for the SCOP/CATH dataset and the full evaluation dataset with weighted HISS scores since no such classifications could be obtained with unweighted HISS scores. Accordingly, weighted HISS scores seem to be the method of choice for comparing a large number of membrane proteins as we intend to do in the following analyses. The differential weighting of helix interactions according to the number of predicted helix interactions allows for a more fine-grained quantification of structural similarity and also seems to have an additional positive effect on classification accuracy as helix interactions with many predicted contacts have a higher chance of being correctly predicted.

**Figure 2:**
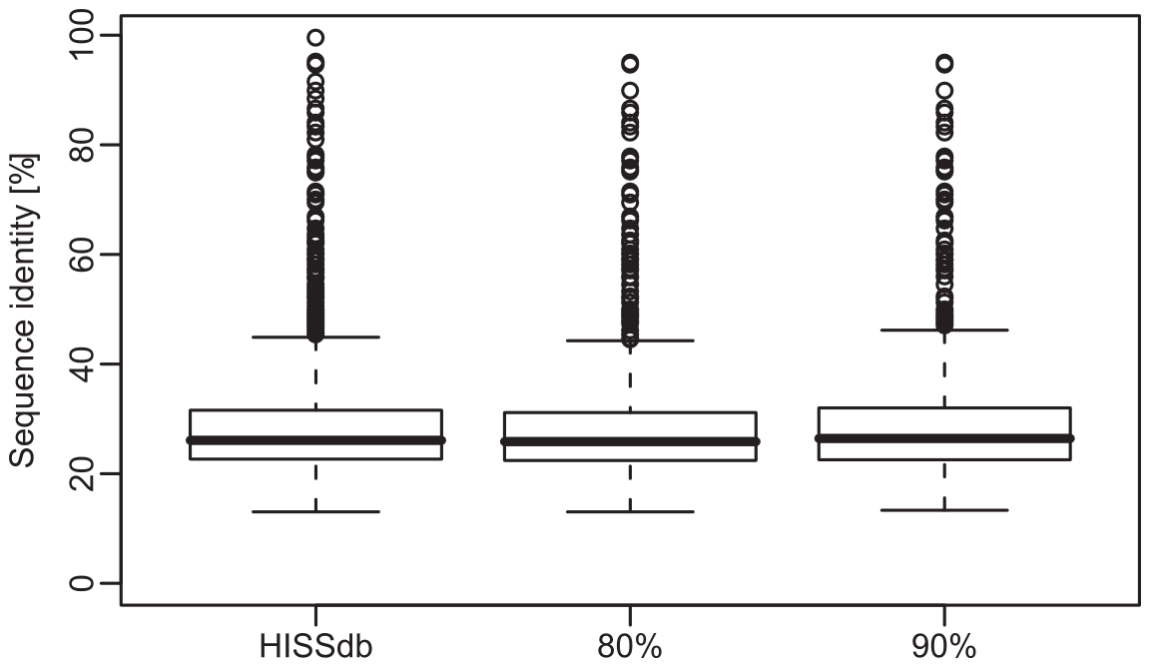
Sequence similarity distribution. Sequence identity among all protein pairs classified to the same architecture within the HISSdb database and the two structural classifications obtained using predicted helix interactions with either 80% specificity or 90% specificity.

Finally, we were interested to find out whether our HISS-based classification of predicted helix architectures captures structural similarity beyond simple sequence similarity or rather tends to identify only those protein pairs as being similar that also have a high sequence identity. To this end, we calculated sequence identities of all protein pairs correctly classified to the same fold based on predicted helix interactions (classifications with weighted HISS scores and 80% or 90% specificity) and compared these identities to the distribution of sequence identities obtained for all protein pairs classified to the same cluster in the HISSdb database (Figure 2). Our method based on predicted helix interactions is able to correctly classify protein pairs over the full range of observed sequence identities and hence all three sequence identity distributions are highly similar. Within HISSdb, the average sequence identity of proteins belonging to the same cluster is 28.7%, which is closely matched by our predicted classifications having average sequence identities of 28.6% (80% specificity) and 29.4% (90% specificity). For the 90% specificity classification, for example, this corresponds to roughly 10% of all correctly classified protein pairs (73 out of 750) having a pairwise sequence identity lower than 20%. For example, the microbial sensory rhodopsin (1xioA) and the beta1-adrenergic receptor from turkey (2vt4A) display a pairwise sequence identity of 17.8% while still belonging to the same CATH fold 1.20.1070. These promising results confirm that structural similarities beyond mere sequence identity can be deduced from predicted helix interaction patterns. While existing classification approaches specifically addressing membrane proteins always use sequence similarity as a major criterion for a common classification [18], [29], the combination of predicted helix interactions and HISS scores offers a completely new possibility of deriving structural similarity originating, for example, from convergent evolution.

### Generation and Large-scale Classification of Predicted Consensus Helix Architectures

In a next step, we refined our method to predict helix interaction patterns by generating consensus helix architectures based on sets of structurally related membrane proteins (SC-clusters) from the CAMPS 2.0 database [15]. In CAMPS 2.0 α–helical membrane proteins are classified into structural families according to sequence similarity, the number of transmembrane helices, and loop length patterns. Although sequence information is not the only feature in the classification, it has a major effect on the clustering result. Thus, it is possible that multiple SC-clusters describe similar structures originating from convergent evolution. By applying the method of consensus helix architectures to SC-clusters we sought to find membrane proteins that share structural similarity, but lack sequence similarity.

We started by determining optimal parameters for building consensus architectures using a set of 28 SC-clusters containing known three-dimensional PDB [20] structures (dataset CAMPS_TEST). Consensus architectures with varying contact thresholds *C* (for NN4 and NN4-D) and consensus thresholds *con* were generated for each of the 28 SC-clusters (as described in *Materials and Methods*) and compared to the observed individual helix architecture of the corresponding structure. Aiming at 80% specificity the best parameter setting was achieved for *C* = 11 (network NN4), *C* = 11 (network NN4-D) and *con* = 0.3 (consensus threshold), yielding a sensitivity of 61.6% (Table 3). For comparison, we also predicted individual helix architectures for the proteins used for building consensus architectures and calculated the average sensitivity and specificity. Similarly, helix architectures were predicted for the PDB proteins themselves and compared to the observed helix architectures. As seen in Table 3 consensus architectures reproduced observed helix arrangements as good or even better as the average helix predictions and the predictions obtained for the PDB proteins. At 80% specificity, consensus architectures were 1.7% more sensitive than the PDB predictions and 2.2% more sensitive than the average predictions.

**Table 3:** Parameter optimization for generation of consensus helix architectures.

Graph type	Contact threshold^a^	Consensus threshold^b^	Accuracy [%]	Sensitivity^c^ [%]	Specificity^d^ [%]
Consensus	C11/C11	0.3	71.8	61.6	80.1
	C12/C14	0.6	69.9	45.4	89.7
PDB^e^	C5/C12	−	70.9	59.9	79.8
	C12/C18	−	69.8	45.4	89.6
Average^f^	C6/C18	−	70.1	59.4	79.5
	C15/C27	−	66.0	41.3	89.5

a Contact threshold (NN4/NN4-D): number of required helix-helix contacts to predict a helix as interacting. NN4 and NN4-D are two versions of the TMHcon software for the prediction of helix-helix contacts.

b Consensus threshold: fraction of individual helix architectures required to contain a helix interaction to transfer it to the consensus architecture.

c Sensitivity: fraction of known interacting helices that can also be found in the predicted architectures.

d Specificity: fraction of known non-interacting helices that are also absent in the predicted architectures.

e PDB: helix architectures derived from known PDB structures were compared with those that were predicted for these PDB proteins.

f Average: helix architectures were predicted for all proteins involved in the consensus architecture and compared with the helix architectures derived from the known PDB structures.

Using the optimal parameters at 80% specificity (*C* = 11 for NN4, *C* = 11 for NN4-D, con = 0.3), we generated consensus architectures for all 431 SC-clusters from the classification dataset (CAMPS_SC) containing proteins with 5 to 15 TMHs. All pairs of consensus architectures representing the same number of TMHs (16,027 pairs in total) were compared to each other using the HISS scoring system. Similar to the clustering of protein pairs (see above), all SC-cluster pairs (represented by their consensus architectures) above a given HISS score threshold were clustered using the MCL algorithm. The resulting clusters representing combinations of SC-clusters were termed HIS ( = helix interaction similarity) clusters. Trying different HISS score thresholds and different MCL inflation values (whereas the inflation value regulates the granularity of the clustering) we calculated the sensitivity and specificity of the HIS clusters with respect to Pfam-A [25] annotations. Sensitivity was defined as the fraction of all protein pairs with the same Pfam annotation that were assigned to the same HIS cluster and specificity as the fraction of all protein pairs with different Pfam annotations assigned to different HIS clusters. The best parameter combination reaching 90% specificity for all 431 SC-clusters corresponds to a HISS score threshold of 0.86 and an inflation value of 2 (Table 4). This combination achieves a sensitivity of almost 52%. However, when we calculated sensitivity and specificity for SC-clusters with members having 5 to 7 TMHs and more than 7 TMHs separately, we found that this parameter setting (0.86/2) is not optimal for both SC-cluster categories. Thus, we derived different parameter settings for the two categories both achieving similar values of sensitivity and specificity. Aiming at 90% specificity, a HISS score threshold of 0.95 and an inflation value of 1.1 were shown to perform best for the first category, achieving almost 55% sensitivity. For the second category, the combination 0.75/1.1 performed with 90% specificity and almost 50% sensitivity (Table 4).

**Table 4:** Parameter optimization for clustering of consensus helix architectures.

SC-cluster dataset^a^	HISS score threshold	Inflation value	Sensitivity^b^ [%]	Specificity^c^ [%]
All	0.70	1.1	67.1	85.3
	0.86	2	51.8	89.5
≤7 TMHs	0.84	5	66.9	78.8
	0.95	1.1	54.9	91.2
>7 TMHs	0.70	1.1	54.2	84.3
	0.75	1.1	49.9	89.5

a All: All SC-clusters from the classification dataset; ≤7 TMHs: SC-clusters with members having up to seven TMHs; >7 TMHs: SC-clusters with members having more than seven TMHs.

b Sensitivity: Fraction of all proteins pairs having the same Pfam annotation that were assigned to the same HIS cluster using the respective HISS score threshold and inflation value.

c Specificity: Fraction of all proteins pairs having different Pfam annotations that were assigned to different HIS clusters using the respective HISS score threshold and inflation value.

Accordingly, we used the parameter combinations 0.95/1.1 (for SC-clusters with up to 7 TMHs) and 0.75/1.1 (for SC-clusters with more than 7 TMHs) for the final clustering of all consensus architectures. The 431 SC-clusters were joined into 151 HIS clusters, whereas 111 of them are singleton clusters (*i.e.* clusters only containing one SC-cluster) and 40 HIS clusters contain two or more SC-clusters (Table 5). Only 15 out of 151 HIS clusters encompass at least one SC-cluster that is associated with known three-dimensional structures. Assuming that each HIS cluster corresponds to a distinct membrane protein fold, 90% of the HIS clusters represent unknown folds, illustrating that the membrane protein structure space is largely unexplored (note that this number refers to membrane proteins with at least five transmembrane helices (see Materials and Methods).

**Table 5:** TMH distribution among SC-clusters and HIS clusters.

Number ofTMHs	Number ofSC-clusters	Number of HIS clusters			Reduction factor^c^
		Singleton^a^	Non-Singleton^b^	Total	
5	97	25	18	43	2.3
6	121	26	8	34	3.6
7	68	28	3	31	2.2
8	24	1	1	2	12.0
9	12	3	1	4	3.0
10	37	16	4	20	1.9
11	27	0	1	1	27.0
12	30	6	1	7	4.3
13	5	0	1	1	5.0
14	8	4	2	6	1.3
15	2	2	0	2	1.0
Total	431	111	40	151	2.9

a HIS cluster containing only one SC-cluster.

b HIS cluster containing two or more SC-clusters.

c Number of SC-clusters divided by total number of HIS clusters.

### Validation of HIS Clusters

#### Comparison with Pfam

Using Pfam-A family and clan annotations of membrane proteins, original (sequence and topology based) SC-clusters and the new helix interaction based clusters (HIS clusters) were compared with each other. It is important to mention at this point that we do not intend to fully reproduce the Pfam clustering. SC-clusters and HIS clusters are designed to represent sets of structurally similar membrane proteins likely to share the same fold, while Pfam is a sequence-based approach and does not consider structural features. Nevertheless, Pfam is used as a reference since Pfam annotations are available for a large majority of membrane proteins (about 70%, data not shown). Among all protein pairs having the same Pfam-A family annotation 66.5% of pairs were also found in the same SC-cluster (sensitivity), while 99.9% of all pairs with different annotations were assigned to different SC-clusters (specificity). Using Pfam clan annotations a sensitivity of 11.3% and a specificity of 100% was observed for SC-clusters. Subsequent clustering of SC-clusters using helix architectures led to significantly improved sensitivity at the cost of a slightly reduced specificity. HIS clustering resulted in a sensitivity of 81.6% (at 94.8% specificity) and 42.0% (at 96.1% specificity) at the family and clan level, respectively. Most importantly, HIS clusters are 30.7% more sensitive than SC-clusters when compared to Pfam clans. Pfam clans are described as a set of related Pfam families that have arisen from a single evolutionary origin [30] and hence tend to group together large, divergent families. Thus, Pfam clans represent a perfect evaluation when investigating the clustering of structurally similar proteins with low sequence similarity. Our results indicate that the further clustering of SC-clusters led to a considerable improvement of the clustering quality, particularly for divergent membrane proteins, as illustrated by the three test cases below.

#### CASE 1: G Protein-Coupled Receptors

**Figure 3:**
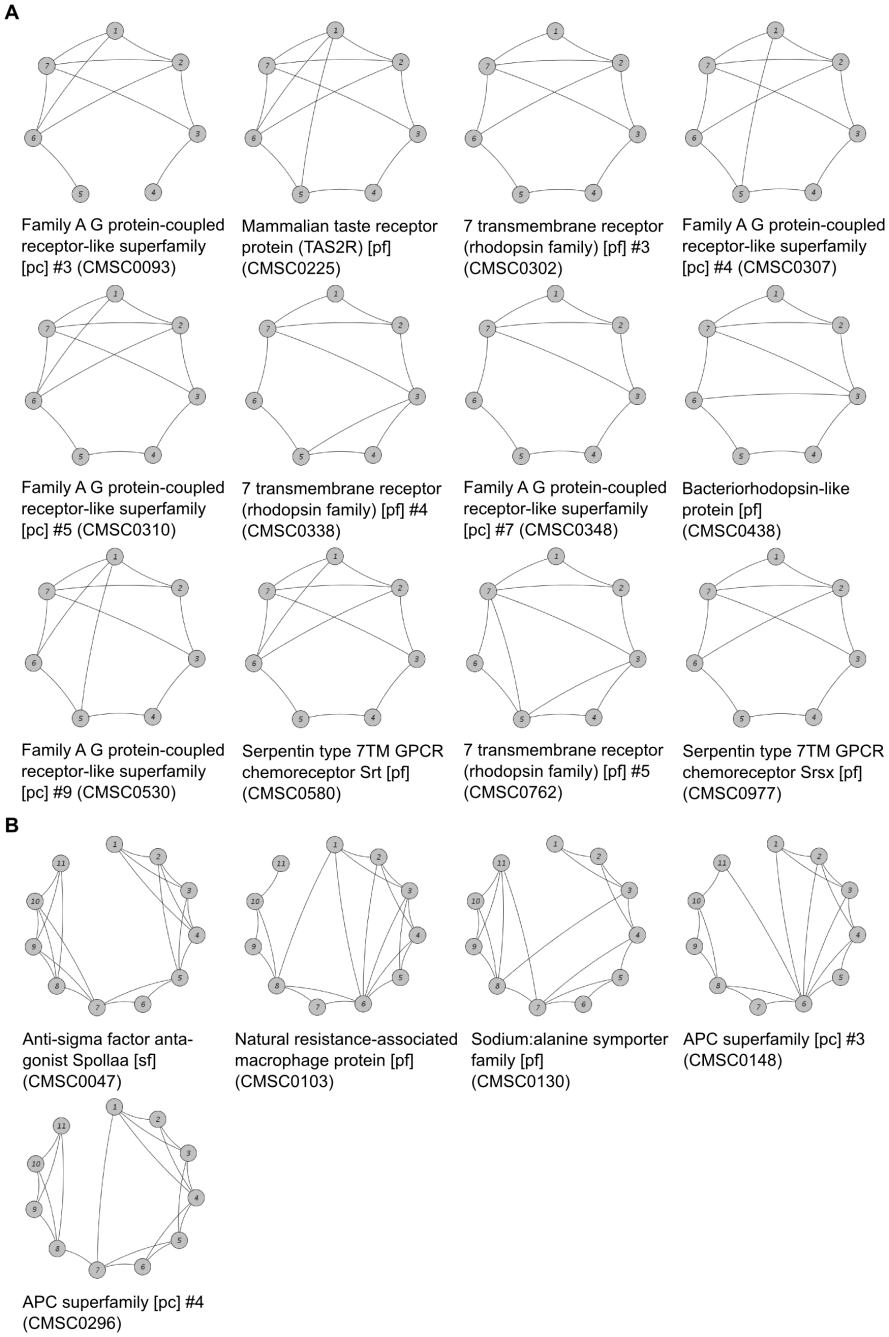
Consensus helix architectures of selected SC-clusters. A: All SC-clusters belong to Pfam clan CL0192 (‘Family A G protein-coupled receptor-like superfamily’) and were joined into the same HIS cluster (CMHIS0006). B: All SC-clusters belong to Pfam clan CL0062 (‘APC superfamily’) and were joined into the same HIS cluster (CMHIS0005). Nodes correspond to transmembrane helices, edges represent helix interactions.

G protein-coupled receptors (GPCRs) are known to be the largest and most diverse protein superfamily in the mammalian genome and are further subdivided into five main families [31]–[33] (with family A being the largest family of GPCRs). All GPCRs share a common structure of a seven transmembrane helix bundle [34], while sequence similarity is rather low among distant GPCRs, which led to several CAMPS SC-clusters containing GPCRs. Clustering based on helix architectures resulted in 12 SC-clusters that all contain members assigned to the Pfam clan CL0192 (‘Family A G protein-coupled receptor-like superfamily’) being grouped into the same HIS cluster due to their similar consensus architectures (Figure 3A). Importantly, protein pairs from different SC-clusters that were merged during this process were found to have an average pairwise sequence identity of 14.1%, which again confirms that our method is able to identify membrane proteins with similar structures lacking significant sequence similarity.

#### Case 2: APC superfamily

Similarly, another five SC-clusters were found that are all linked with Pfam clan CL0062 (‘APC superfamily’) and are now classified to the same HIS cluster (Figure 3B). While GPCRs are only present in eukaryotes, amino acid/polyamine/organocation (APC) transporters are numerous in all domains of life. As in the previous case, the average pairwise sequence identity between protein pairs originating from different SC-clusters (joined into the same HIS cluster) was found to be very low (14.5%).

#### CASE 3: SC-clusters with similar 3D structures

**Figure 4:**
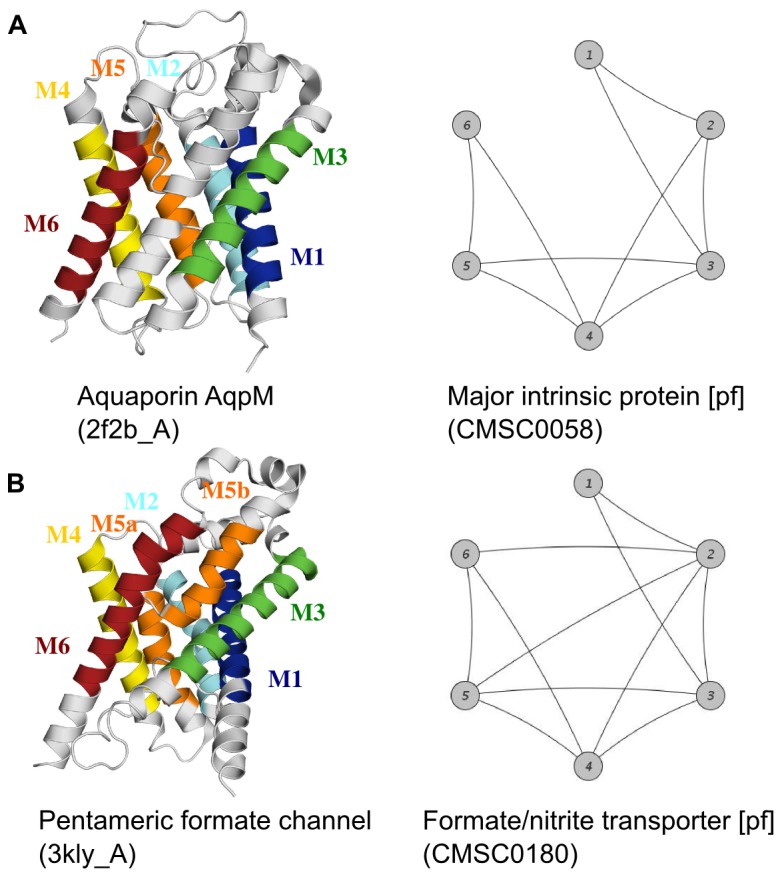
Example of two SC-clusters that were joined together. Both SC-clusters contain structures with a very similar transmembrane helix packing. (A) Left panel: Representative structure (PDB code: 2f2b, chain A) of SC-cluster CMSC0058. Right panel: Consensus helix architecture for SC-cluster CMSC0058. (B) Left panel: Representative structure (PDB code: 3 kly, chain A) of SC-cluster CMSC0180. Right panel: Consensus helix architecture for SC-cluster CMSC0180. Both structures contain six transmembrane helices (M1–M6) colored differently. The fifth helix of 3 kly_A is interrupted (M5a, M5b). Nodes correspond to transmembrane helices, edges represent helix interactions. Transmembrane helix coordinates were extracted from PDBTM [16].

The last case is special as it shows two SC-clusters associated with known 3D structures being similar to each other and joined into the same HIS cluster (Figure 4). SC-Clusters CMSC0058 and CMSC0180 contain the archaeal aquaporin AqpM (2f2b, chain A) and the bacterial formate channel (3 kly, chain A), respectively. By comparing the two structures using DaliLite [35] we observed a high degree of structural similarity (*Z*-score: 17.6). While a *Z*-score of at least 2.0 indicates a common fold, a *Z*-score above 20 means that two structures are true homologs (see the DaliLite help file at http://www.ebi.ac.uk/Tools/dalilite). At the same time, the sequence identity of the two channels is only 15.3%. It is interesting to note that both structures are also classified to the same CATH [12] fold (‘Glycerol uptake facilitator protein’) and to the same OPM [36] superfamily (‘Major Intrinsic Protein (MIP)/FNT superfamily’). Furthermore, Theobald and Miller also revealed that the two channels share a common structural fold in the absence of sequence similarity raising questions about the evolution of membrane proteins [37].

Taken together, our results clearly indicate that using predicted helix architectures it is possible to identify structurally similar membrane proteins lacking sequence similarity. This ability can aid in discerning distant evolutionary relationships between membrane proteins and in organizing the membrane protein fold space, as demonstrated below.

### Exploring the Membrane Protein Fold Space

#### Distribution of helix architectures across TMH classes

**Figure 5:**
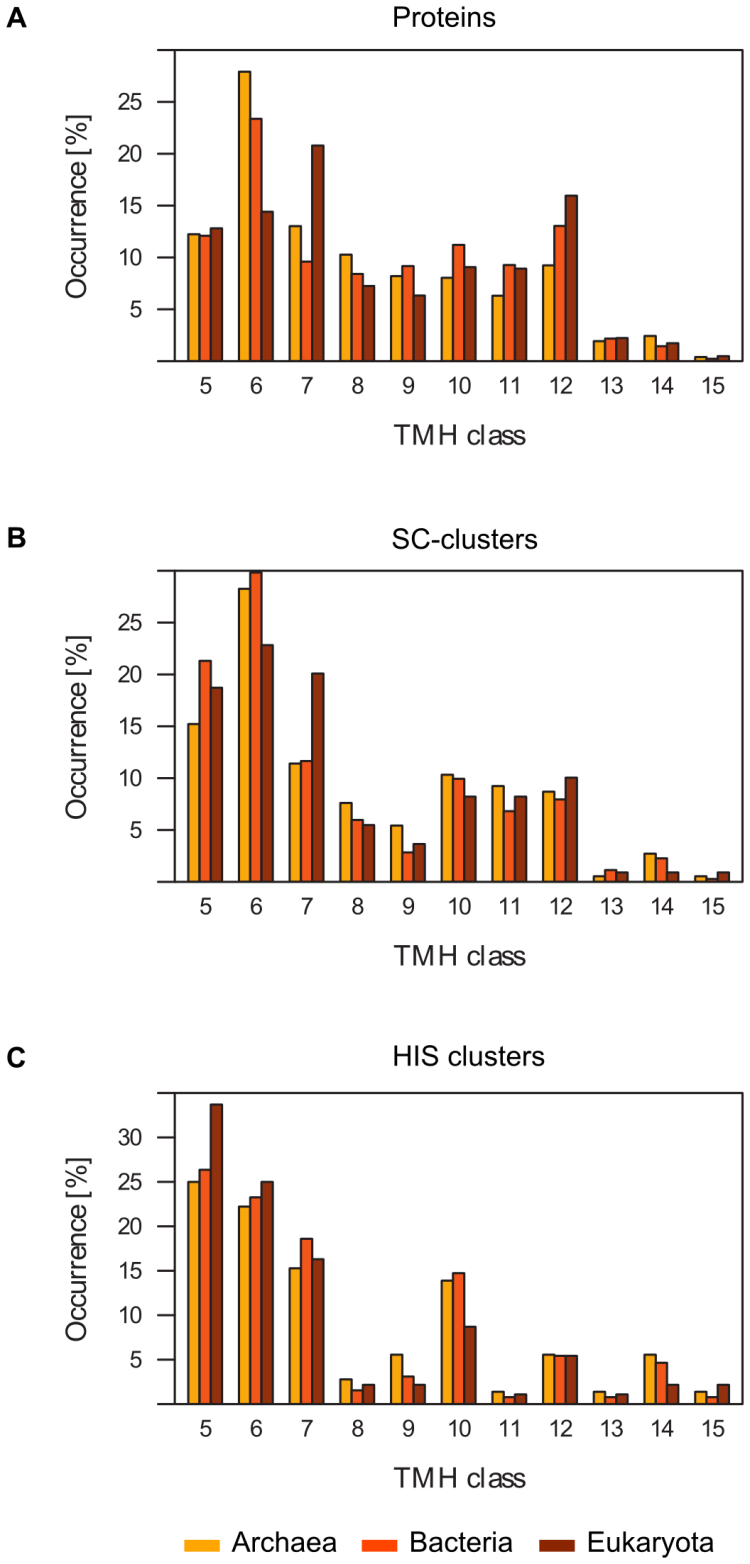
Occurrence of TMH classes among individual proteins, SC-clusters and HIS clusters. A: Percentage of proteins with a certain number of TMHs. Percentage of SC-clusters (B) and HIS clusters (C) with a certain representative TMH number.

After validating our new classification of predicted membrane protein architectures (i.e. HIS clusters) against Pfam clans we investigated how many distinct protein architectures could be observed with a given number of transmembrane helices and how this distribution differed from corresponding distributions considering the original SC-clusters or individual membrane proteins (Figure 5, Table 5). In 1998 Jones analyzed the patterns of occurrence of transmembrane topologies [38]. This and other genome-wide analyses [39]–[43] showed that proteins with 6 and 12 TMHs, such as small-molecule transporters, sugar transporters and ABC transporters, are predominant in uni-cellular organisms. In contrast, proteins with 7 TMHs are abundant in *C. elegans* and human due to the high abundance of G-protein coupled receptors. For the membrane proteins in our classification dataset (CAMPS_SC) we could observe similar trends (see Figure 5A and Table S1 in the Supporting Information), except for the 12 TMH class which is more abundant in eukaryotes than in prokaryotes (Eukaryota: 16.0%, Archaea: 9.2%, Bacteria: 13.0%). A likely explanation from this deviation from earlier analyses is that they were based on not more than four eukaryotic genomes while the CAMPS database incorporates 134 eukaryotic genomes in total [15].

Similarly, we also investigated the distribution of TMH classes among our HIS clusters in comparison to the original SC-clusters (see Figures 5B and 5C and Table S1 in the Supporting Information). While the distribution at the protein level reflects the mere abundance of different TMH classes, the distribution at the cluster level (especially the HIS cluster level) rather displays their structural diversity. As can be seen in Figures 5A and 5B, the distribution of SC-clusters is highly similar to the protein distribution with slight differences for the 8, 9 and 12 TMH class. While proteins with 8 and 9 TMHs are almost equally abundant as proteins with 10 TMHs, SC-clusters with 8 and 9 TMH proteins are less frequent than those involving the 10 TMH topology. In contrast to these minor differences, the distribution of HIS clusters differs significantly from both the protein and the SC-cluster distribution: while the 6 TMH class is abundant among prokaryotic SC-clusters and the 7 TMH class among eukaryotic SC-clusters, the number of HIS clusters decreases from 5TMH to 7TMH in all superkingdoms. Furthermore, the fraction of HIS clusters with 8, 11 and 12 TMH proteins was clearly reduced in comparison to their respective frequency in the SC-cluster and protein distribution. Overall we observe that proteins are more likely to have similar helix interaction patterns and therefore also similar predicted folds the more helices they have (hence the reduction in the number of different architectures), which is rather surprising as an increase in the number of helices would potentially open up a much larger combinatorial space for different possible helix interaction patterns.

The abundance of proteins with 8 to 12 TMHs is rather homogeneous (see Figure 5A), while HIS clusters with the 10 and 12 TMH topology are more frequent than HIS clusters with, for example, the 11 TMH topology (see Figure 5C) suggesting that these membrane protein classes are structurally more diverse. It is known from previous publications that internal gene duplications are a very common mechanism in membrane protein evolution [2], [44]–[49]. According to these studies proteins with 10 and 12 TMHs seem to have evolved through a complete gene duplication. Therefore, we speculate that proteins with 10 and 12 TMHs show a higher structural diversity as they originated from proteins with 5 and 6 TMHs that themselves are distributed among many different HIS clusters. Similarly, we suggest that for the same reason proteins with 14 TMHs adopt more diverse structures than proteins with 13 TMHs.

#### Structural similarity of transporter families with 8 TMH and 11 TMH

We investigated more closely the 8 and 11 TMH classes that appear to make the largest contribution to the almost 3-fold decrease in the number of protein clusters after the clustering based on helix architecture similarity (Table 5). Thus, 23 out of 24 SC-clusters with members having eight TMHs were joined into one HIS cluster. Different transporter proteins including ABC transporters, nickel transporters, NADH dehydrogenases and P-type ATPases, as well as many proteins of unknown function were grouped together. Hence, it seems that these transporters have a common structural core, a structural similarity that could not be revealed using the SC-clustering approach. In fact, previous studies based on hydropathy profile analysis also revealed structural similarities between different families of secondary transporters not related in amino acid sequence, indicating distant evolutionary relationships [50]–[53]. Therefore, we hypothesize that structural similarity is likely to be found in other transporter families as well, and our HIS cluster (joining 23 SC-clusters) is one more case of structurally related transporters that arose either by divergent or convergent evolution [54]. The second remaining HIS cluster containing 8 TMH proteins is a singleton cluster comprising only one SC-cluster (CAMPS code CMSC0049). Given that CMSC0049 includes proteins of unknown function, it might be an interesting target for structural genomics.

All 27 SC-clusters of the 11 TMH class were joined into a single HIS cluster (Table 5). Again, most of the SC-clusters represent different transporters, such as sulfate, ammonium, metal ion and amino acid transporters, as well as sodium/alanine and sodium/glutamate transporters. Furthermore, the grouped SC-clusters are linked with different Pfam clans (CL0062 ‘APC superfamily’, CL0064 ‘CPA/AT transporter superfamily’, CL0182 ‘IT superfamily’). Taken together, for the special case of 8 and 11 TMH proteins, we assume that (almost) all SC-clusters were combined into one HIS cluster because the considered transporter families share a common structural core.

#### Functional diversity of membrane protein classes and HIS clusters

In a final analysis we investigated how the structural diversity observed within our predicted membrane protein architectures correlates with functional diversity among the classified proteins. To this end we performed a GO (Gene Ontology) term enrichment analysis for both TMH classes and HIS clusters in order to find out whether TMH classes associated with many HIS clusters are also associated with a large number of enriched GO terms. The highest number of distinct GO terms was found for proteins with 5, 6 and 7 TMHs (having 6 to 7 terms; see Table S2 and Table S3 in the Supporting Information). In this case functional diversity seems to imply structural diversity. The same applies to the 10 TMH and 14 TMH classes (5 and 4 terms, respectively) as compared to the remaining classes in the range of 8 to 15 TMHs. However, it has to be noted that the difference in the number of distinct GO terms is often too subtle in order to draw clear conclusions regarding the correlation between structural and functional diversity. For example, the 13 TMH and 14 TMH classes differ remarkably in their structural variability (according to the number of different HIS clusters), but only slightly in their functional variability (3 and 4 enriched GO terms, respectively). The number of GO terms for the 13 TMH class drops down to 2, if we consider the terms ‘transport’ and ‘transmembrane transport’ as only one enriched term, as the second is just a more detailed description of the first one.

We used enriched GO terms to draw conclusions about the functional diversity of different membrane protein classes. However, we are aware of the fact that this approach is not necessarily straightforward. First, one protein can be associated with multiple GO terms (multi-functional protein). Second, one HIS cluster can be linked with multiple GO terms (multi-functional cluster). Therefore, the statement that the more GO terms can be found the higher the functional diversity might be misleading. To investigate the effect of multi-functional proteins we looked at the protein annotations and searched for GO terms that frequently occur in combination with other GO terms. This was found to occur in three cases: i) when proteins with 5, 9, and 10 TMHs were annotated with the GO term ‘cell division’, the ‘cell cycle’ annotation was available as well, ii) the same applies to the combination ‘protein targeting’ and ‘transport’ concerning the 6 TMH class, and iii) the annotations ‘transport’, ‘transmembrane transport’ and ‘response to stress’ also occurred in combination affecting the 11, 12 and 13 TMH classes.

Similarly, to analyze the presence of multi-functional HIS clusters, we performed a separate GO enrichment analysis (see Table S4 in the Supporting Information). Except for HIS clusters with 15 TMHs, one or more significantly (i.e. *P*-value≤0.05) enriched GO terms could be found for all HIS clusters. Compared to the enrichment analysis at the protein level additional GO terms were found to be enriched that were, however, not considered for further analyses. In total, we found seven HIS clusters to contain multiple enriched GO terms (CMHIS0048, CMHIS0029, CMHIS0010, CMHIS0001, CMHIS0006, CMHIS0004, CMHIS0003) whereas the terms that were earlier found to occur frequently in combination were considered as only one term. Additionally, we also observed that several HIS clusters from the same TMH class are associated with the same GO terms. For example, four clusters (CMHIS0137, CMHIS0143, CMHIS0130, CMHIS0006) of the 7 TMH class contain the term ‘signal transduction’. Taking into account the effects of multi-functional proteins, multi-functional HIS clusters and HIS clusters with the same functional annotations we conclude that the structural diversity of HIS clusters can be explained by functional diversity, at least to some extent.

## Conclusions

Structure classification of membrane proteins is hampered by the paucity of structural data, necessitating alternative approaches to explore the membrane protein fold space. Here, we present a new structural classification approach for α-helical membrane proteins using predicted helix architectures. In a first step we evaluated how well predicted helix architectures reflect structural similarity between membrane proteins. Subsequently consensus helix architectures were generated for selected SC-clusters from our CAMPS database and then SC-clusters with similar interaction patterns, not necessarily related at the sequence level, were joined into so called HIS clusters. Finally, HIS clusters were further investigated regarding their structural and functional properties.

Predicted helix architectures were not only shown to successfully recognize similar membrane protein structures, achieving a sensitivity of 65% at 90% specificity when compared to SCOP and CATH, but also to be able to identify membrane proteins sharing the same fold but almost no sequence similarity. Organizing membrane proteins according to their helix interaction patterns has shed some new light on the structure space of membrane proteins. In particular, we found that although in large membrane proteins the number of possible arrangements of transmembrane helices is virtually unlimited proteins with 8 and more helices are distributed over considerably fewer different architectures than proteins with up to 7 helices. This finding suggests that in the course of membrane protein evolution a limited number of folding arrangements have been repeatedly re-used. This effect seems to be especially prevalent in the case of transporter proteins with either 8 or 11 transmembrane helices, which all seem to share a common helix interaction pattern.

Apart from using predicted helix architectures for structural classification we believe that they can also find application in predicting domain boundaries. Since interactions within domains are more frequent than between domains it may be possible to delineate domain boundaries by searching for tightly connected sub-graphs on helix-helix interaction graphs. Such sequence-based domain recognition would allow to further fine-tune membrane protein structure classification.

## Supporting Information

Table S1 **Distribution of transmembrane helices among individual proteins, SC-clusters and HIS clusters.**
(DOC)

Table S2 **Enriched GO terms in TMH classes. For each TMH class (i.e. set of proteins with a respective number of transmembrane helices), the number of proteins that are annotated with the respective enriched GO term is given.** If no number is given, the respective count is zero. All listed GO terms are enriched with a P-value≤0.05.
(DOC)

Table S3 **Significantly enriched GO terms for protein class level enrichment analysis.**
(DOC)

Table S4 **Significantly enriched GO terms for cluster level enrichment analysis.**
(DOC)
