# Supplementary material for: Classification of α-Helical Membrane Proteins Using Predicted Helix Architectures
Source: PLoS One. 2013 Oct 25;8(10):e77491. doi: 10.1371/journal.pone.0077491 (PMC3808409; doi:10.1371/journal.pone.0077491)
Supplement: Table S1 — Distribution of transmembrane helices among individual proteins, SC-clusters and HIS clusters. (DOC) [file pone.0077491.s001.doc]

**Table S1.** Distribution of transmembrane helices among individual proteins, SC-clusters and HIS clusters.

|  | Number of TMHs | | | | | | | | | | |
| --- | --- | --- | --- | --- | --- | --- | --- | --- | --- | --- | --- |
|  | 5 | 6 | 7 | 8 | 9 | 10 | 11 | 12 | 13 | 14 | 15 |
| **Number of proteins with the given number of TMHs** | | | | | | | | | | | |
| Archaea | 615 | 1,402 | 654 | 516 | 412 | 404 | 317 | 464 | 97 | 122 | 20 |
| Bacteria | 13,770 | 26,622 | 10,939 | 9,585 | 10,449 | 12,774 | 10,556 | 14,852 | 2,482 | 1,635 | 263 |
| Eukaryota | 3,231 | 3,636 | 5,245 | 1,829 | 1,598 | 2,289 | 2,250 | 4,026 | 562 | 437 | 122 |
| **Number of SC-clusters containing members with the given number of TMHs** | | | | | | | | | | | |
| Archaea | 28 | 52 | 21 | 14 | 10 | 19 | 17 | 16 | 1 | 5 | 1 |
| Bacteria | 75 | 105 | 41 | 21 | 10 | 35 | 24 | 28 | 4 | 8 | 1 |
| Eukaryota | 41 | 50 | 44 | 12 | 8 | 18 | 18 | 22 | 2 | 2 | 2 |
| **Number of HIS clusters containing members with the given number of TMHs** | | | | | | | | | | | |
| Archaea | 18 | 16 | 11 | 2 | 4 | 10 | 1 | 4 | 1 | 4 | 1 |
| Bacteria | 34 | 30 | 24 | 2 | 4 | 19 | 1 | 7 | 1 | 6 | 1 |
| Eukaryota | 31 | 23 | 15 | 2 | 2 | 8 | 1 | 5 | 1 | 2 | 2 |
