# Supplementary material for: Classification of α-Helical Membrane Proteins Using Predicted Helix Architectures
Source: PLoS One. 2013 Oct 25;8(10):e77491. doi: 10.1371/journal.pone.0077491 (PMC3808409; doi:10.1371/journal.pone.0077491)
Supplement: Table S2 — Enriched GO terms in TMH classes. For each TMH class (i.e. set of proteins with a respective number of transmembrane helices), the number of proteins that are annotated with the respective enriched GO term is given. If no number is given, the respective count is zero. All listed GO terms are enriched with a P-value≤0.05. (DOC) [file pone.0077491.s002.doc]

1. **Table S2.** Enriched GO terms in TMH classes. For each TMH class (i.e. set of proteins with a respective number of transmembrane helices), the number of proteins that are annotated with the respective enriched GO term is given. If no number is given, the respective count is zero. All listed GO terms are enriched with a P-value ≤ 0.05.

|  | TMH class | | | | | | | | | | | TMH classes^c^ |
| --- | --- | --- | --- | --- | --- | --- | --- | --- | --- | --- | --- | --- |
|  | 5 | 6 | 7 | 8 | 9 | 10 | 11 | 12 | 13 | 14 | 15 |
| Size^a^ | 15,627 | 29,439 | 14,358 | 10,806 | 11,403 | 14,145 | 11,896 | 17,277 | 2,805 | 1,880 | 324 |
| Annotations^b^ | 4,283 | 13,741 | 4,472 | 3,997 | 4,702 | 5,763 | 5,436 | 11,389 | 1,495 | 556 | 67 |
| GO term | Number of proteins in TMH class with annotated GO term | | | | | | | | | | |
| Carbohydrate metabolic process |  |  |  |  |  |  | 10 |  |  |  |  | 1 |
| Cell cycle | 137 |  |  |  | 252 | 427 |  |  |  |  |  | 3 |
| Cell differentiation |  |  |  | 6 |  |  |  |  |  |  |  | 1 |
| Cell division | 140 |  |  |  | 107 | 244 |  |  |  |  |  | 3 |
| Cell proliferation |  |  | 5 |  |  |  |  |  |  |  |  | 1 |
| Cellular component assembly |  | 132 | 56 | 152 |  |  |  |  |  |  |  | 3 |
| Cellular nitrogen compound metabolic process |  |  |  |  |  |  |  |  |  | 4 |  | 1 |
| Embryo development |  |  |  |  |  | 5 |  |  |  |  |  | 1 |
| Lipid metabolic process | 22 | 45 | 20 |  |  |  |  |  |  |  |  | 3 |
| Locomotion |  |  | 12 |  |  |  |  |  |  |  |  | 1 |
| Photosynthesis |  |  |  |  |  | 3 |  |  |  |  |  | 1 |
| Protein complex assembly |  | 132 | 56 | 152 |  |  |  |  |  |  |  | 3 |
| Protein targeting | 240 | 456 |  |  |  | 235 |  |  |  |  |  | 3 |
| Reproduction |  |  | 6 |  |  |  |  |  |  |  |  | 1 |
| Response to stress |  |  |  |  |  |  |  | 34 | 32 |  |  | 2 |
| Signal transduction | 153 | 275 | 200 |  |  |  |  |  |  | 53 |  | 4 |
| Small molecule metabolic process |  |  |  |  |  |  |  |  |  | 2 |  | 1 |
| Transmembrane transport |  |  |  |  |  |  | 2,707 | 8,866 | 1,221 | 391 | 40 | 5 |
| Transport |  | 11,303 |  |  |  |  | 5,068 | 11,236 | 1,429 |  |  | 4 |
| Vesicle-mediated transport | 11 |  |  |  |  |  |  |  |  |  |  | 1 |
| GO terms^d^ | 6 | 6 | 7 | 3 | 2 | 5 | 3 | 3 | 3 | 4 | 1 |  |

1. ^a^ Number of proteins in the TMH class.
2. ^b^ Number of proteins in the TMH class with GO annotations.
3. ^c^ Number of TMH classes that contain this enriched GO term.

^d^ Number of enriched GO terms in this TMH class.
