# Supplementary material for: Classification of α-Helical Membrane Proteins Using Predicted Helix Architectures
Source: PLoS One. 2013 Oct 25;8(10):e77491. doi: 10.1371/journal.pone.0077491 (PMC3808409; doi:10.1371/journal.pone.0077491)
Supplement: Table S3 — Significantly enriched GO terms for protein class level enrichment analysis. (DOC) [file pone.0077491.s003.doc]

**Table S3.** Significantly enriched GO terms for protein class level enrichment analysis.

| GO term | Description | Study count | Population count | Adjusted *P*-value |
| --- | --- | --- | --- | --- |
| **5 TMH** | | | | |
| GO:0006605 | Protein targeting | 240 | 1128 | 1.14E-46 |
| GO:0006629 | Lipid metabolic process | 22 | 97 | 4.20E-05 |
| GO:0007049 | Cell cycle | 137 | 961 | 2.08E-13 |
| GO:0007165 | Signal transduction | 153 | 708 | 3.44E-34 |
| GO:0016192 | Vesicle-mediated transport | 11 | 13 | 3.25E-10 |
| GO:0051301 | Cell division | 140 | 588 | 4.43E-36 |
| **6 TMH** | | | | |
| GO:0006461 | Protein complex assembly | 132 | 390 | 1.59E-04 |
| GO:0006605 | Protein targeting | 456 | 1,128 | 7.86E-28 |
| GO:0006629 | Lipid metabolic process | 45 | 97 | 3.86E-05 |
| GO:0006810 | Transport | 11,303 | 47,363 | 4.07E-03 |
| GO:0007165 | Signal transduction | 275 | 708 | 5.59E-18 |
| GO:0022607 | Cellular component assembly | 132 | 390 | 1.59E-04 |
| **7 TMH** | | | | |
| GO:0000003 | Reproduction | 6 | 11 | 3.14E-03 |
| GO:0006461 | Protein complex assembly | 56 | 390 | 3.43E-04 |
| GO:0006629 | Lipid metabolic process | 20 | 97 | 2.25E-03 |
| GO:0007165 | Signal transduction | 200 | 708 | 5.23E-58 |
| GO:0008283 | Cell proliferation | 5 | 7 | 2.26E-03 |
| GO:0022607 | Cellular component assembly | 56 | 390 | 3.43E-04 |
| GO:0040011 | Locomotion | 12 | 15 | 7.92E-10 |
| **8 TMH** | | | | |
| GO:0006461 | Protein complex assembly | 152 | 390 | 8.14E-68 |
| GO:0022607 | Cellular component assembly | 152 | 390 | 8.14E-68 |
| GO:0030154 | Cell differentiation | 6 | 15 | 1.85E-02 |
| **9 TMH** | | | | |
| GO:0007049 | Cell cycle | 252 | 961 | 7.80E-57 |
| GO:0051301 | Cell division | 107 | 588 | 1.07E-11 |
| **10 TMH** | | | | |
| GO:0006605 | Protein targeting | 235 | 1,128 | 5.46E-26 |
| GO:0007049 | Cell cycle | 427 | 961 | 1.61E-167 |
| GO:0009790 | Embryo development | 5 | 7 | 7.19E-03 |
| GO:0015979 | Photosynthesis | 3 | 3 | 3.93E-02 |
| GO:0051301 | Cell division | 244 | 588 | 7.71E-87 |
| **11 TMH** | | | | |
| GO:0005975 | Carbohydrate metabolic process | 10 | 16 | 2.10E-05 |
| GO:0006810 | Transport | 5,068 | 47,363 | 3.93E-41 |
| GO:0055085 | Transmembrane transport | 2,707 | 18,420 | 2.36E-107 |
| **12 TMH** | | | | |
| GO:0006810 | Transport | 11,236 | 47,363 | 2.09E-185 |
| GO:0006950 | Response to stress | 34 | 88 | 2.06E-02 |
| GO:0055085 | Transmembrane transport | 8,866 | 18,420 | 0 |
| **13 TMH** | | | | |
| GO:0006810 | Transport | 1,429 | 47,363 | 3.40E-15 |
| GO:0006950 | Response to stress | 32 | 88 | 2.26E-25 |
| GO:0055085 | Transmembrane transport | 1,221 | 18,420 | 2.11E-297 |
| **14 TMH** | | | | |
| GO:0007165 | Signal transduction | 53 | 708 | 1.54E-26 |
| GO:0034641 | Cellular nitrogen compound metabolic process | 4 | 8 | 2.25E-05 |
| GO:0044281 | Small molecule metabolic process | 2 | 5 | 2.72E-2 |
| GO:0055085 | Transmembrane transport | 391 | 18,420 | 6.43E-75 |
| **15 TMH** | | | | |
| GO:0055085 | Transmembrane transport | 40 | 18,420 | 1.57E-03 |
