# Supplementary material for: Classification of α-Helical Membrane Proteins Using Predicted Helix Architectures
Source: PLoS One. 2013 Oct 25;8(10):e77491. doi: 10.1371/journal.pone.0077491 (PMC3808409; doi:10.1371/journal.pone.0077491)
Supplement: Table S4 — Significantly enriched GO terms for cluster level enrichment analysis. (DOC) [file pone.0077491.s004.doc]

**Table S4.** Significantly enriched GO terms for cluster level enrichment analysis.

| Cluster | GO term^a^ | Description | Study count | Population count | Adjusted *P*-value |
| --- | --- | --- | --- | --- | --- |
|  | **5 TMH** | | | | |
| CMHIS0074 | GO:0006629* | Lipid metabolic process | 1 | 64 | 1.26E-02 |
| CMHIS0054 | GO:0016192* | Vesicle-mediated transport | 1 | 10 | 4.50E-03 |
|  | GO:0030154 | Cell differentiation | 3 | 7 | 5.43E-09 |
| CMHIS0034 | GO:0007165* | Signal transduction | 2 | 518 | 1.68E-02 |
| CMHIS0055 | GO:0016192* | Vesicle-mediated transport | 6 | 10 | 2.99E-21 |
| CMHIS0048 | GO:0007049* | Cell cycle | 132 | 562 | 5.48E-220 |
|  | GO:0007165* | Signal transduction | 9 | 518 | 5.60E-03 |
|  | GO:0051301* | Cell division | 132 | 401 | 1.23E-242 |
| CMHIS0063 | GO:0006457 | Protein folding | 1 | 5 | 1.05E-02 |
| CMHIS0058 | GO:0051301* | Cell division | 3 | 401 | 4.50E-05 |
| CMHIS0042 | GO:0007165* | Signal transduction | 13 | 518 | 9.72E-20 |
| CMHIS0085 | GO:0007067 | Mitosis | 1 | 1 | 2.60E-03 |
|  | GO:0007155 | Cell adhesion | 2 | 5 | 7.77E-06 |
| CMHIS0017 | GO:0040011 | Locomotion | 1 | 14 | 4.96E-02 |
| CMHIS0059 | GO:0006950 | Response to stress | 2 | 59 | 2.88E-04 |
|  | GO:0007568 | Aging | 2 | 4 | 1.01E-06 |
|  | GO:0008283 | Cell proliferation | 1 | 3 | 5.10E-03 |
| CMHIS0029 | GO:0007165* | Signal transduction | 21 | 518 | 2.70E-29 |
|  | GO:0016192* | Vesicle-mediated transport | 3 | 10 | 1.83E-09 |
| CMHIS0100 | GO:0006629* | Lipid metabolic process | 10 | 64 | 6.48E-27 |
|  | GO:0008219 | Cell death | 1 | 3 | 7.90E-03 |
|  | **6 TMH** | | | | |
| CMHIS0096 | GO:0040007 | Growth | 1 | 12 | 7.90E-03 |
|  | GO:0040011 | Locomotion | 1 | 14 | 9.20E-03 |
| CMHIS0010 | GO:0006810* | Transport | 1,205 | 28,687 | 1.97E-04 |
|  | GO:0007165* | Signal transduction | 37 | 518 | 1.52E-02 |
|  | GO:0055085 | Transmembrane transport | 1,205 | 12,033 | 0 |
| CMHIS0014 | GO:0006810* | Transport | 1,360 | 28,687 | 1.24E-35 |
| CMHIS0020 | GO:0006457 | Protein folding | 1 | 5 | 9.90E-03 |
| CMHIS0092 | GO:0006629* | Lipid metabolic process | 40 | 64 | 4.90E-114 |
| CMHIS0050 | GO:0007165* | Signal transduction | 95 | 518 | 5.25E-172 |
| CMHIS0015 | GO:0006412 | Translation | 3 | 5 | 1.32E-02 |
|  | GO:0006810* | Transport | 1,540 | 28,687 | 3.93E-40 |
| CMHIS0012 | GO:0006810* | Transport | 1,260 | 28,687 | 5.87E-33 |
| CMHIS0024 | GO:0006605* | Protein targeting | 301 | 691 | 0 |
|  | GO:0006810* | Transport | 308 | 28,687 | 1.33E-07 |
| CMHIS0001 | GO:0006461* | Protein complex assembly | 130 | 286 | 1.78E-41 |
|  | GO:0022607* | Cellular component assembly | 130 | 286 | 1.87E-41 |
| CMHIS0071 | GO:0006605* | Protein targeting | 16 | 691 | 2.87E-24 |
| CMHIS0128 | GO:0007165* | Signal transduction | 25 | 518 | 6.71E-45 |
| CMHIS0011 | GO:0006810* | Transport | 1,631 | 28,687 | 6.21E-43 |
| CMHIS0135 | GO:0007165* | Signal transduction | 16 | 518 | 2.37E-28 |
| CMHIS0104 | GO:0055085 | Transmembrane transport | 13 | 12,033 | 9.50E-03 |
|  | **7 TMH** | | | | |
| CMHIS0056 | GO:0006810 | Transport | 114 | 28,687 | 6.00E-03 |
| CMHIS0137 | GO:0007165* | Signal transduction | 2 | 518 | 1.40E-03 |
| CMHIS0143 | GO:0007165* | Signal transduction | 5 | 518 | 2.81E-09 |
| CMHIS0049 | GO:0055085 | Transmembrane transport | 278 | 12,033 | 2.15E-105 |
|  | GO:0006810 | Transport | 278 | 28,687 | 1.35E-05 |
| CMHIS0130 | GO:0007165* | Signal transduction | 2 | 518 | 1.40E-03 |
| CMHIS0006 | GO:0000003* | Reproduction | 6 | 8 | 5.92E-10 |
|  | GO:0006629* | Lipid metabolic process | 8 | 64 | 5.01E-06 |
|  | GO:0007165* | Signal transduction | 174 | 518 | 5.12E-241 |
|  | GO:0040007 | Growth | 5 | 12 | 1.77E-06 |
|  | GO:0040011* | Locomotion | 12 | 14 | 1.04E-21 |
| CMHIS0147 | GO:0055085 | Transmembrane transport | 8 | 12,033 | 9.60E-03 |
|  | **8 TMH** | | | | |
| CMHIS0004 | GO:0006461* | Protein complex assembly | 152 | 286 | 1.63E-110 |
|  | GO:0022607* | Cellular component assembly | 152 | 286 | 1.63E-110 |
| CMHIS0018 | GO:0040007 | Growth | 1 | 12 | 2.13E-02 |
|  | **9 TMH** | | | | |
| CMHIS0007 | GO:0006810 | Transport | 1,265 | 28,687 | 3.86E-17 |
|  | **10 TMH** | | | | |
| CMHIS0022 | GO:0007049* | Cell cycle | 423 | 562 | 0 |
|  | GO:0051301* | Cell division | 239 | 401 | 0 |
| CMHIS0039 | GO:0006810 | Transport | 248 | 28,687 | 4.70E-06 |
|  | GO:0055085 | Transmembrane transport | 248 | 12,033 | 6.61E-94 |
| CMHIS0021 | GO:0006457 | Protein folding | 2 | 5 | 5.44E-06 |
|  | GO:0007049* | Cell cycle | 4 | 562 | 4.63E-05 |
|  | GO:0051301* | Cell division | 4 | 401 | 1.21E-05 |
| CMHIS0003 | GO:0006605* | Protein targeting | 235 | 691 | 2.93E-84 |
|  | GO:0006810 | Transport | 2,120 | 28,687 | 1.74E-16 |
|  | GO:0009790* | Embryo development | 5 | 6 | 1.85E-04 |
| CMHIS0118 | GO:0040007 | Growth | 1 | 12 | 2.00E-03 |
|  | **11 TMH** | | | | |
| CMHIS0005 | GO:0006810* | Transport | 1,102 | 28,687 | 1.27E-05 |
|  | GO:0055086* | Transmembrane transport | 566 | 12,033 | 2.49E-09 |
|  | **12 TMH** | | | | |
| CMHIS0088 | GO:0055085* | Transmembrane transport | 44 | 12,033 | 1.43E-16 |
| CMHIS0008 | GO:0006810* | Transport | 1,524 | 28,687 | 8.69E-40 |
| CMHIS0002 | GO:0006810* | Transport | 8,168 | 28,687 | 8.73E-228 |
|  | GO:0055085* | Transmembrane transport | 8,141 | 12,033 | 0 |
| CMHIS0037 | GO:0006810* | Transport | 346 | 28,687 | 7.54E-09 |
|  | **13 TMH** | | | | |
| CMHIS0016 | GO:0006810* | Transport | 570 | 28,687 | 2.29E-14 |
|  | GO:0006950* | Response to stress | 28 | 59 | 8.59E-32 |
|  | GO:0055085* | Transmembrane transport | 570 | 12,033 | 3.43E-218 |
|  | **14 TMH** | | | | |
| CMHIS0013 | GO:0055085* | Transmembrane transport | 67 | 12,033 | 3.77E-25 |
| CMHIS0097 | GO:0007165* | Signal transduction | 53 | 518 | 5.87E-95 |

^a^ Asterisks indicate GO terms that were already found to be enriched by the protein class level enrichment analysis (see Table S2).
